# Novel Homozygous CYP27B1 Gene Mutation in Vitamin D-Dependent Rickets Type 1A (VDDR1A) Disorder: A Case Report

**DOI:** 10.3389/fendo.2022.862022

**Published:** 2022-05-18

**Authors:** Doua Khalid Al Homyani, Shahad Khalid Alhemaiani

**Affiliations:** ^1^ Endocrine Division, Department of Pediatrics, Taif Children Hospital, Taif, Saudi Arabia; ^2^ Radiology Division, King Abdulaziz Specialist Hospital, Taif, Saudi Arabia

**Keywords:** VDDR1A, CYP27B1 gene, dependent rickets, 1,25-dihydroxyvitamin D, 25 hydroxyvitamin D

## Abstract

**Background:**

Vitamin D-dependent rickets type 1A (VDDR1A) rickets is an uncommon kind of rickets that affects both boys and girls. Children with mutations are normal at birth and present at around 6 months to 2 years of age with symptoms. When suspected, genetic testing is required to confirm the diagnosis

**Case Presentation:**

This is a case report of VDDR1A in a 4-year-old boy who presented with delayed growth, inability to stand, and rachitic bone deformities. The diagnosis was reached by anthropometric measurement, bone profile, and radiological studies, then confirmed by genetic testing, which revealed a homozygous pathogenic variant in the CYP27B1 gene. He was treated with Vitamin-D (alfacalcidol) and oral calcium.

**Conclusion:**

VDDR1A is caused by a mutation in the CYP27B1 gene, which impairs the 1 hydroxylase enzyme, which compromises vitamin-D production.

## Introduction

Rickets is a disease that causes weak or fragile bones in children (1). Rachitic symptoms include slowed growth, bowed legs, bone pain, prominent brow, and sleeping difficulties (1, 2). Long-term Bone fractures, muscle spasms, hypertrophic cardiomyopathy and an improperly curved spine are all possible complications (1–3).

Rickets is a disease that is prevalent in the Middle East, Africa, and Asia (4). It is uncommon in the United States and Europe, with the exception of a few minority communities (1, 4). It usually begins in childhood, between the ages of three and eighteen months (2, 4). Males and females have the same illness rates (1). Rickets is a rare disease in developed areas (incidence of less than 1 in 200,000) (4).

There are several subtypes of rickets, knowledge of categorization of the type rickets is essential for prompt diagnosis and proper management.

Vitamin D (calciferol) is made up of two fat-soluble pro-hormones that are physiologically inactive. The first is ergocalciferol (vitamin D2), which is created from ergosterol following exposure to ultraviolet (UV) radiation, and the second is cholecalciferol (vitamin D3), which is derived from animal tissues and 7-dehydrocholesterol, which is formed in human skin by UV rays (5). For activation, both forms require a two-step hydroxylation at the 25^th^ and 1^st^ carbons. where hepatic 25-hydroxylase converts vitamin D to 25-hydroxyvitamin D (25-OHD). The second stage takes place mostly in the kidney, where 25-OHD is further hydroxylated by mitochondrial vitamin D 1-hydroxylase to produce the physiologically active hormone 1,25-dihydroxyvitamin D (1,25-(OH)2D), which attaches to its nuclear receptor and performs its biological functions (5–7). The biologically active form of vitamin D, 1,25-(OH)2D, is important for calcium homeostasis and bone metabolism, as well as cell proliferation and differentiation in a range of tissues (5, 7, 8). Renal 1,25-OH2D synthesis from its precursor 25-OHD is a rate-limiting step that is tightly regulated by existing serum concentrations of 1,25(OH)2D, parathyroid hormone (PTH), fibroblast growth factor-23 (FGF-23), calcium, and phosphate concentrations, with renal 1-hydroxylase being stimulated by PTH, hypophosphatemia, or hypocalcemia and inhibited by FGF-23 (8).

Rickets can be caused by four unusual genetic abnormalities in vitamin D metabolism. The first is a deficit in 1-hydroxylase, which is also known as vitamin D dependent rickets type 1A. (VDDR1A). Type 1B deficit is caused by a specific mutation in the CYP2R1 gene that results in a lack of 25-hydroxylase (VDDR1B). Vitamin D resistant rickets (VDRR), also known as to X-linked dominant hypophophatemic rickets. This form is more usually referred to as VDDR type 2A, is caused by a malfunctioning vitamin D receptor (VDR) (VDDR2A). VDDR2B is a rare kind of rickets caused by aberrant production of a hormone response element-binding protein that prevents VDR from performing its normal function (9–12).

To date, 88 changes in the CYP27B1 gene have been discovered using data from the Human Gene Mutation Database (HGMD). These mutations affect all exons of the gene and generally consist of missense and nonsense changes, as well as splicing site modifications, insertions, deletions, and duplications [HGMD], http://www.hgmd.cf.ac.uk/ac/index.php (13).

VDDR1A (vitamin D dependent rickets type 1A) is an autosomal recessive disease caused by mutations in the 1-hydroxylase gene (CYB27B1). Genetic analysis is vital for getting a precise diagnosis because it can be mistaken with nutritional rickets and hypophosphatemic rickets (14). Hypotonia, muscle weakness, inability to walk, growth failure, and rickets-like radiological abnormalities are all symptoms of VDDR1A (15, 16). We present a case of VDDR1A in a 4-year-old child with sluggish growth, inability to stand, and bending of both legs in this study.

## Case Presentation

A 4-year-old boy child presented with complaints of slow growth, inability to stand, and bowing of the legs. He had gotten vitamin D3 400 IU once daily for six months before to this presentation from an orthopedic clinic in another hospital, but his symptoms had not improved.

His birth record stated that he was born healthy and weighed 3,000 grams (average body weight of full-term ranging from 2500 g to 3500 g) (17).

While his family history revealed that he was the fifth child of consanguineous parents, fourth degree relative (first cousins), the rest of their children are fine. His parents were in good condition and had no concerns with metabolic bone disease. In terms of his feeding history, he spent his first year on Similac formula. In terms of his developmental history, he grew his first teeth at the age of one year (typically, a baby sprout first teeth between the ages of six and ten months) and began walking at the age of eighteen months (normally a baby starts walking between 8-18 months). His development and weight remained stable at 8 months, and he lost the capacity to crawl and stand correctly, despite the fact that he had no history of seizures or tetany.

**Figure 1:**
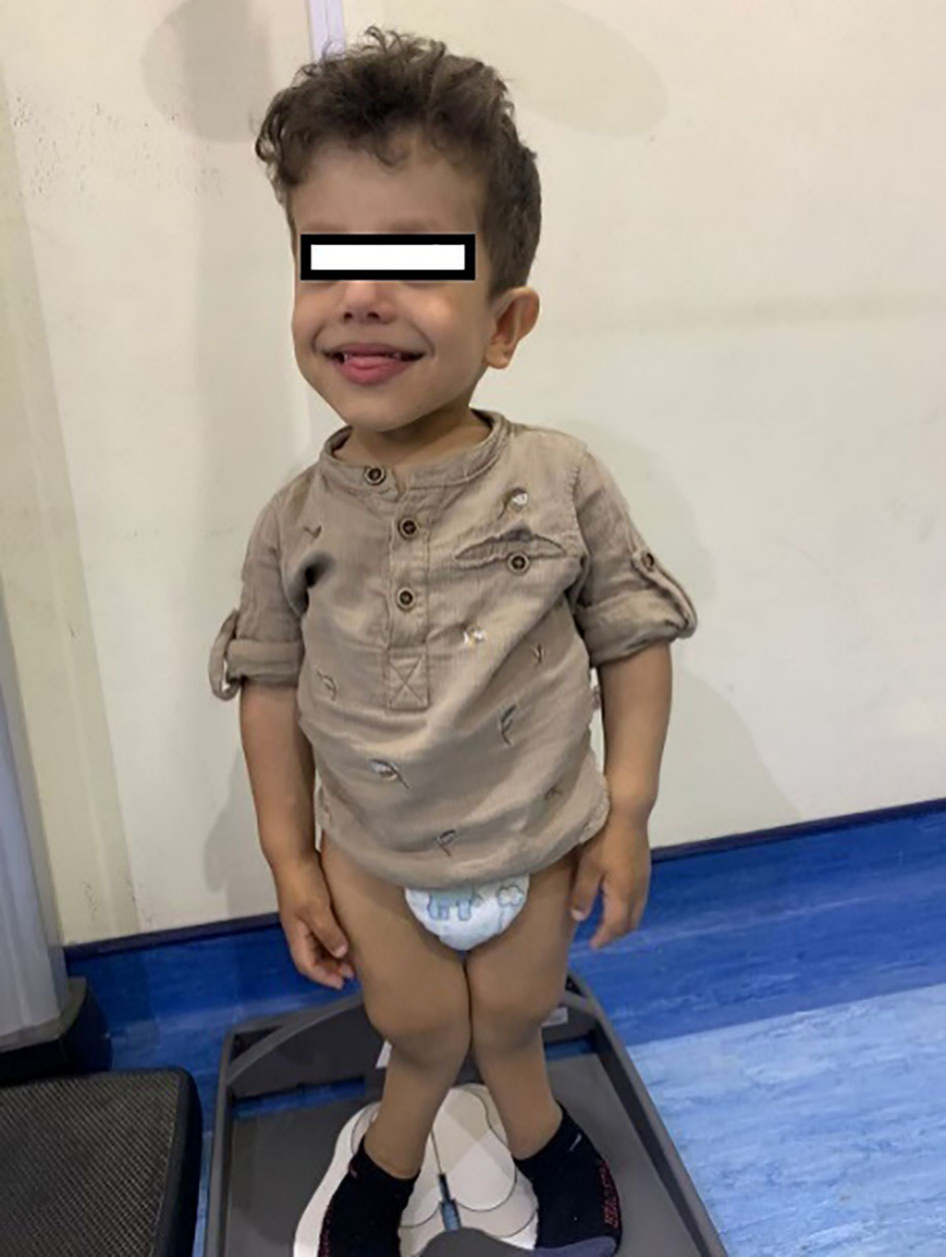
Leg bowing and genu valgum.

**Figure 2:**
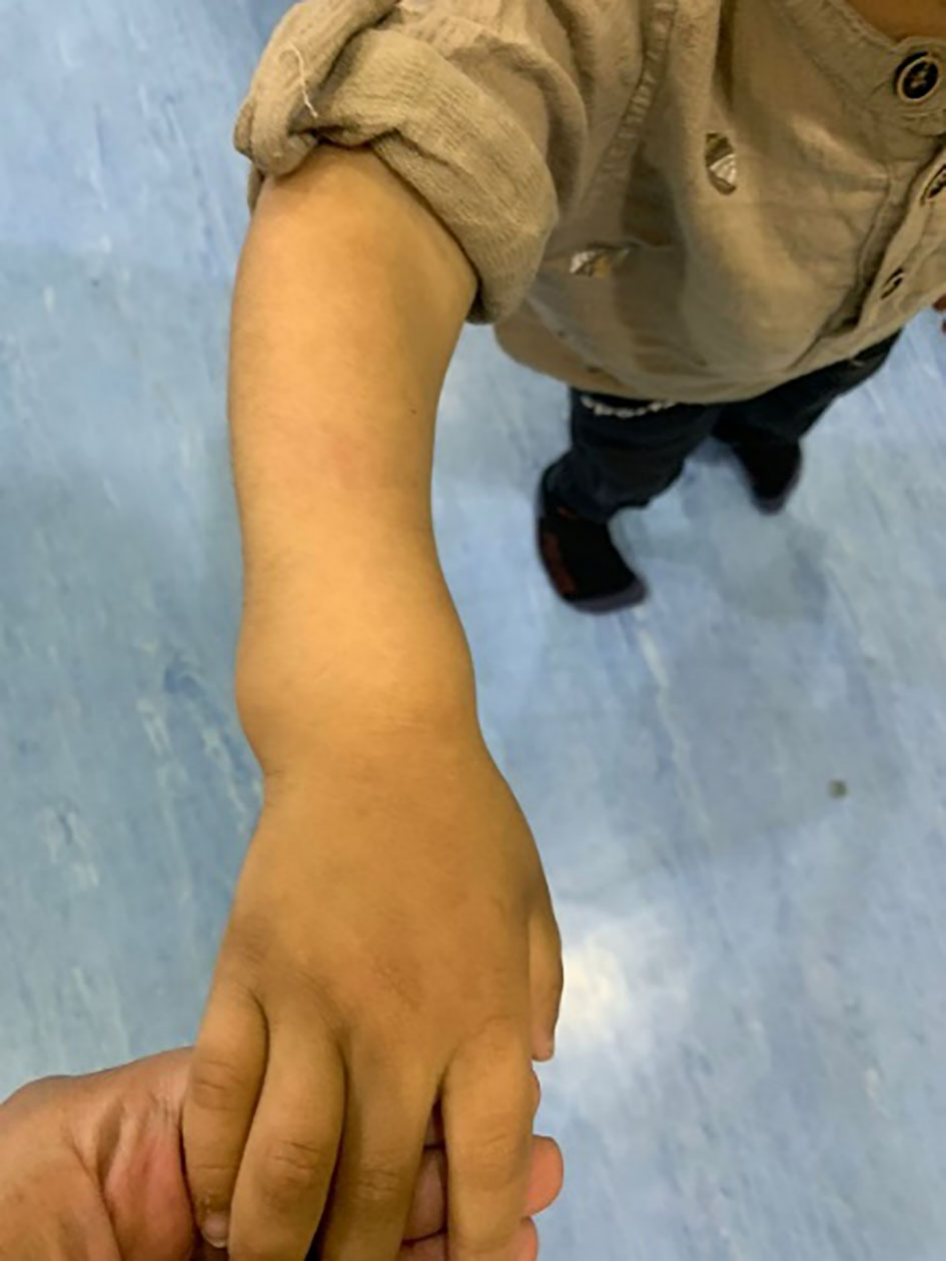
Wrist joint widening.

**Figure 3:**
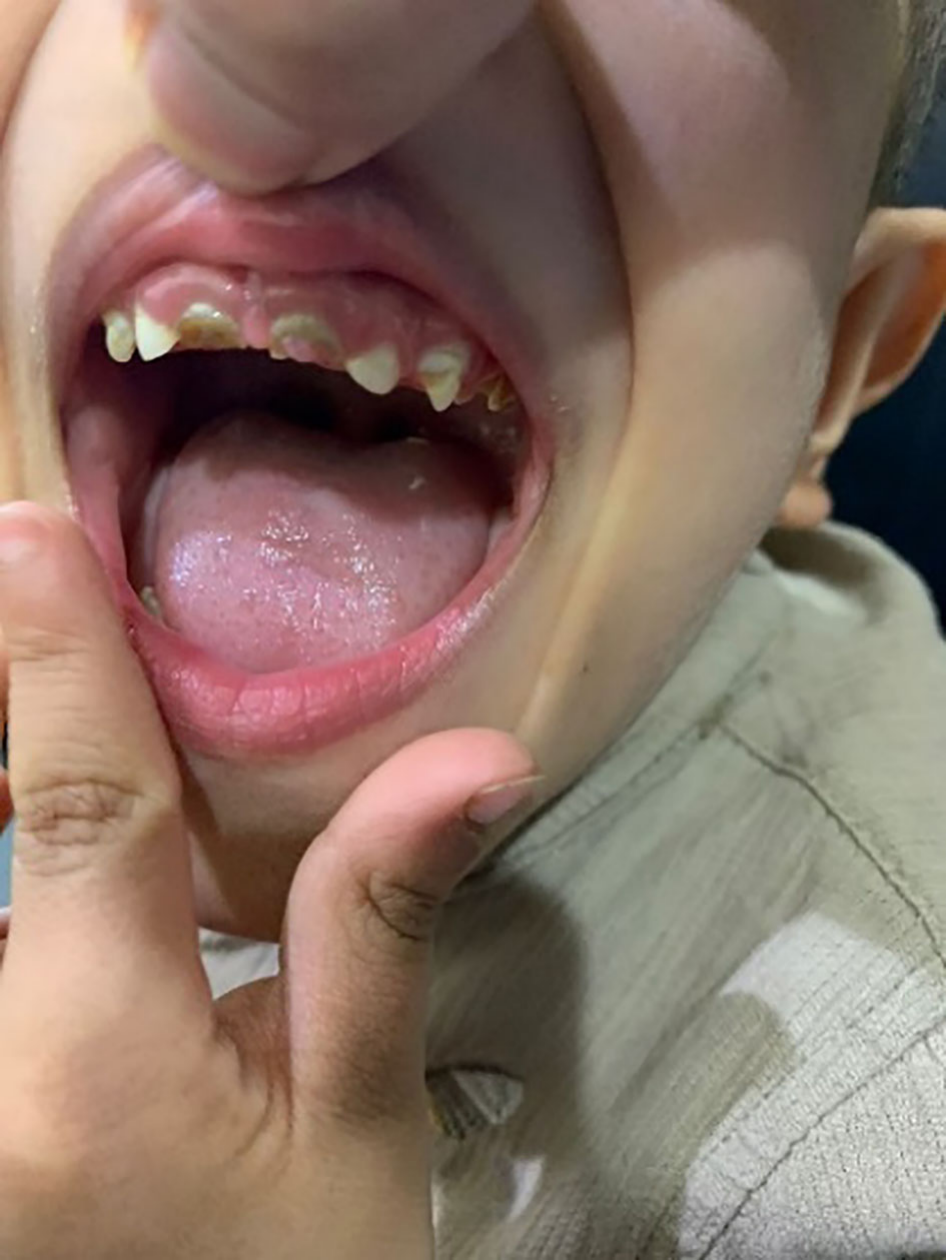
Dental caries in the upper incisors and molar teeth.

**Figure 4:**
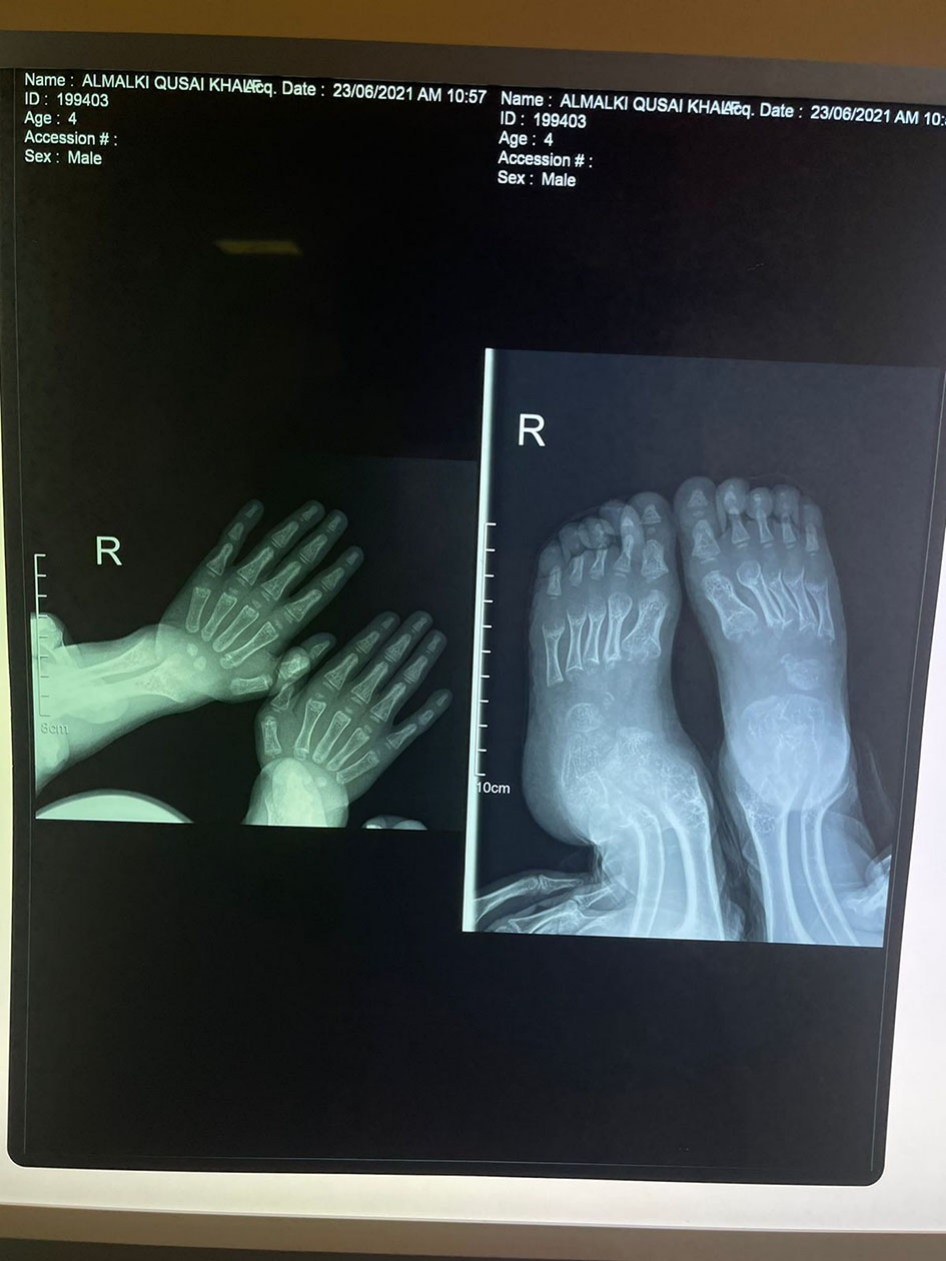
Fraying, splaying, widening and cupping of the metaphyseal endplates of upper and lower limb bones.

**Figure 5:**
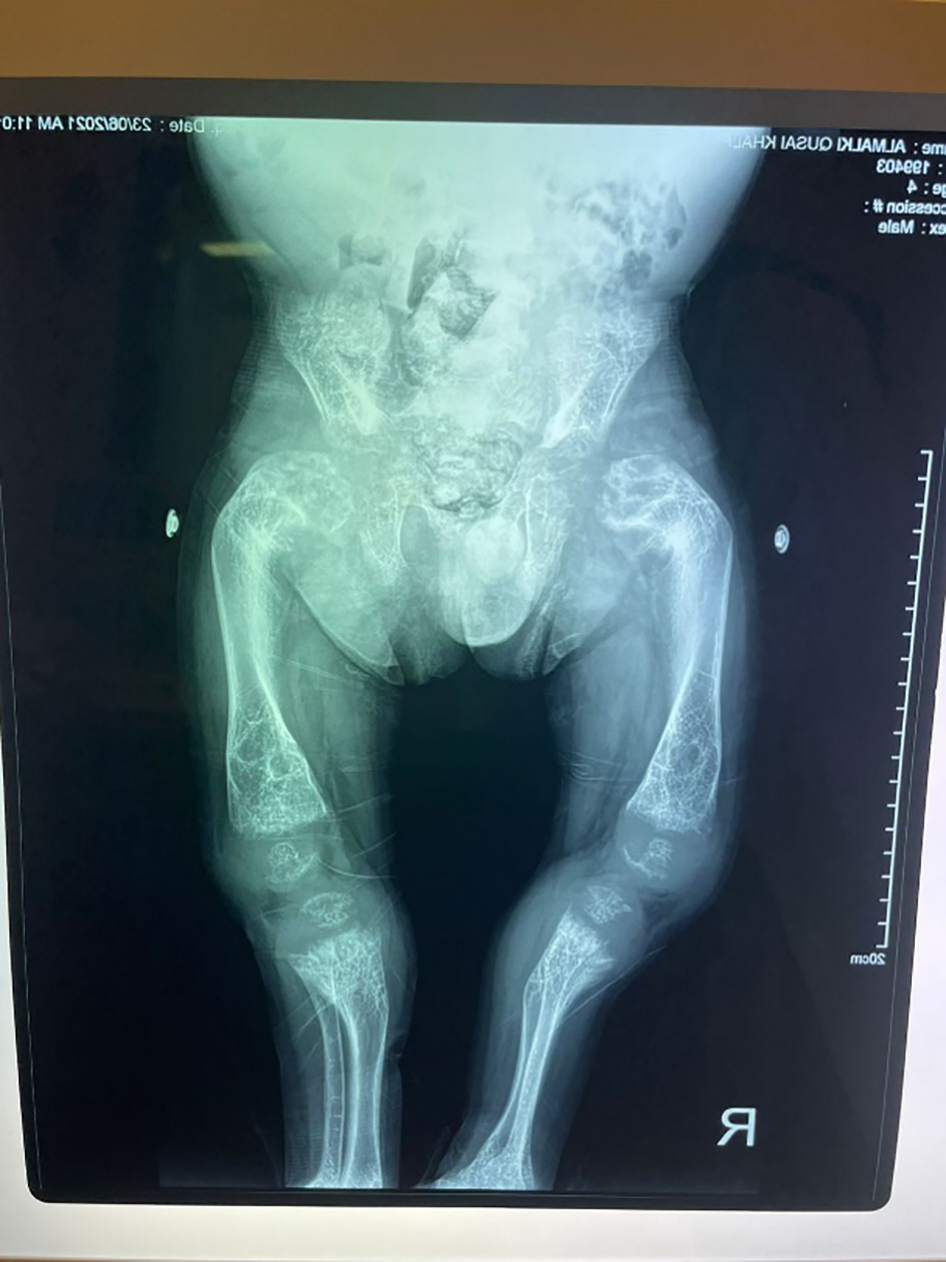
Marked bowing of both femurs and tibiae.

**Figure 6:**
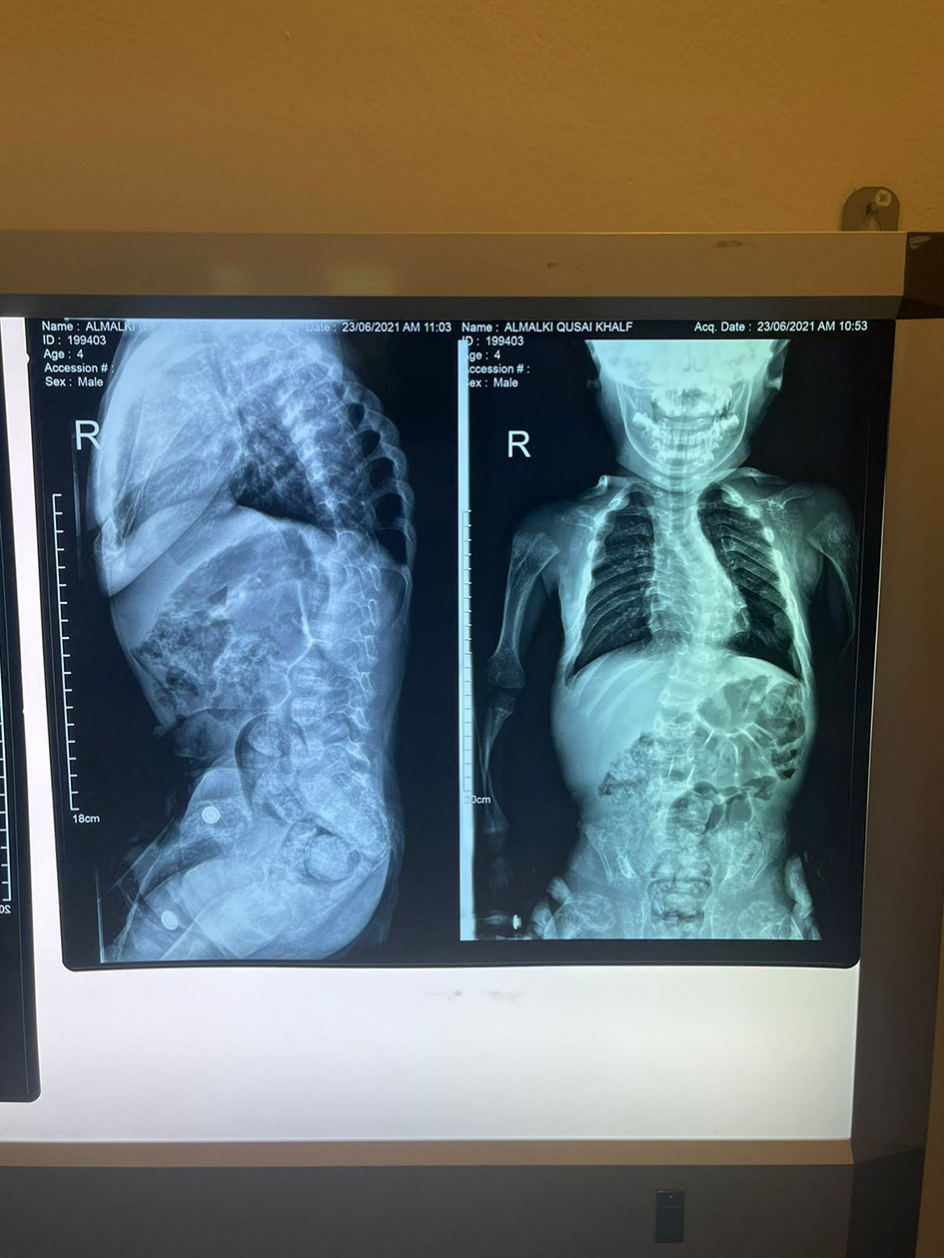
Marked scoliotic deformity and marked osteopenic changes. The final diagnosis by a genetic study showed a homozygous likely pathogenic variant was identified in exon 8 of the CYP27B1 gene: c.1375C>T. p.(R459C). Accordingly, this finding was consistent with the genetic diagnosis of autosomal recessive Vitamin D dependent rickets type 1A (VDDR1A). After six months of treatment with oral calcium (elemental 50mg/kg/d) and alfacalcidol (1α-hydroxycolecalciferol) 50 ng/kg/d. He has minor radiological healed, but his clinical characteristics have not changed significantly as a result of severe bone abnormalities caused by a late diagnosis. Except for a high urine calcium creatinine ratio, his biochemical tests were normal (**Table 1**), and the treatment was adjusted accordingly. The radiological features take much longer than the biochemistry to return to normal. Surgery could also be necessary later on after medical therapy has failed.

His anthropometric measurements revealed a disproportionately short stature (**Figure 1**), with a height of 77 cm, which was below the 1st percentile (93.6 cm), a body weight of 11 kg, which was below the 1st percentile (12.2 kg), and a borderline head circumference of 48 cm, which was over the 5th percentile (47.8 cm). Frontal bossing, rachitic rosary, wrist widening (**Figure 2**), scoliosis with a bent spine, and bilateral genu valgum (**Figure 1**), in addition to muscle hypotonia, were all visible signs of rickets. Dental caries were also discovered in the upper incisors and molar teeth (**Figure 3**). The following are the findings of the laboratory investigations: Serum Ca: 2.02 mmol/l, serum phosphorus: 0.84 mmol/l, Serum alkaline phosphatase: 1881IU/L, albumin 42 g/l, serum 25-OH vitamin D: 175 nmol/l, Serum Parathyroid hormone (PTH) level: 65.1 pmol/L, serum 1,25 OH D 122.4 pmol/L, Ratio of tubular maximum reabsorption rate of phosphate to glomerular filtration rate: TmP/GFR:1.5 mmol/L, urine calcium (mg/dL):urine creatinine (mmol/mmol) ratio: 0.14. Complete blood count, liver function tests and renal panel were normal. Thyroid-stimulating hormone (TSH): 5 mU/L and Free thyroxin 4 (FT4): 2.5 umol/L (**Table 1**). The radiological skeletal survey showed generalized osteopenia. Scoliosis with a curve on the left side (Cobb angle 44°) was present. The classic radiological features of rickets (**Figure 4**), broadening, cupping, and fraying of metaphyseal ends of upper and lower limb bones, were present (**Figure 5**). There was also a ‘rugger jersey’ appearance to the spine (**Figure 6**). This describes the prominent endplate densities at multiple contiguous vertebral levels to produce an alternating sclerotic-lucent–sclerotic appearance, that mimics the horizontal stripes of a rugby jersey. Vertebral insufficiency fractures of the lower dorsal vertebra were also present. Renal ultrasonography revealed no nephrocalcinosis and the kidney echogenicity was normal.

**Table 1:** Results of blood investigation.

	Value	3 months after treatment	6 months after treatment	Normal range
Ca mmol/L	2.02	2.2	2.3	2.1-2.6
Po4 mmol/L	0.84	1.5	1.7	0.7-1.3
Mg mmol/L	0.76	0.94		0.7-1.2
ALP IU/L	1881	803	348	145-420
PTH pmol/L	65.1	9.8	1.7	1-6.8
Urine Ca : Creatinine mmol/mmol	0.14	0.6	1	0.04-0.7
25 D –OH vitamin pmol/L	175	111.6		50-125
1.25 OH D pmol/l	122.4			36-216
TmP/GFR mmol/L	1.53			1.05-1.78
TSH mU/L	5			0.6-5.5
Free T4 umol/L	2.5			1-2.7
Albumin g/l	42			38-54

## Discussion

VDDR1A (vitamin D dependent rickets type 1A) is an autosomal recessive condition caused by mutations in the 25-OHD 1-hydroxylase gene (CYB27B1). CYB27B1 is made up of nine exons and is about 5 megabytes in size. Hypotonia, muscle weakness, inability to walk, growth failure, and rickets-like radiological abnormalities are all symptoms of VDDR1A. Hypocalcemia, high blood levels of alkaline phosphatase (ALP), and PTH low or normal levels of 1,25-(OH)2D despite normal or increasing concentrations of 25-OHD are common laboratory results (16). Aminoaciduria and hyperchloremic acidosis are common in VDDR1 patients as consequent upon the secondary hyperparathyroidism (7).

The human CYP27B1 gene for 1 hydroxylase, which is one of the most essential enzymes in vitamin D production and represents a regulatory step. The hydroxylation of 25(OH) D to the active form 1,25 (OH)2 D is carried out by this enzyme (18). CYP27B1 was first localized to chromosome 12q13-14 by linkage studies in 1990, and then cloned in mice and then humans in 1997 (17). There are nine exons and eight introns in it (19).

The reduction of 1-hydroxylase activity caused by CYP27B1 mutations necessitates the use of calcitriol (1,25-dihydroxycolecalciferol) or Alfacalcidol (1α-hydroxycholecalciferol) to correct the clinical and laboratory abnormalities (20).

There isn’t much of a link between genetics and phenotype. Mild VDDR1A instances have been documented with various mutations (E189G, E189K, and L343F), notwithstanding the absence of enzymatic activity (21). A G102E mutation was also discovered in a big family with a wide range of illness manifestations (22). These differences in clinical presentation suggest that additional factors may be at play, influencing the disease’s severity.

VDDR1A might be mistaken for other diseases such as hypophosphatemic rickets since they both have low serum phosphate levels, hypocalcemia, and a dramatically increased alkaline phosphatase. A high PTH level, on the other hand, suggests the presence of VDDR1A and aids in the distinction between the two diseases.

Alfacalcidol or calcitriol are effective treatments for VDDR1A instances. Clinical, biochemical, and radiological symptoms are entirely resolved at the physiologic dose of calcitriol (15-30 ng/kg/d) or Alfacalcidol (30-50 ng/kg/d). Stopping medication for a short time, on the other hand, was linked to a recurrence of biochemical abnormalities. Treatment must be continued for the rest of one’s life in order to treat hypocalcemia without hypercalciuria, enhance muscle function, and mend skeletal symptoms (22).

## Conclusion

VDDR1A is caused by a mutation in the CYP27B1 gene, which affects the 1 hydroxylase enzyme, which is involved in vitamin D metabolism. Anthropometric evaluation, bone profile, and radiographic examinations are used to make the diagnosis, as well as a history of rickets that does not respond to vitamin D treatment and confirmation gene testing.

There are few reports of VDDR-1A with normal levels of 1,25-(OH) 2 vitamin D in the clinical setting; the purpose of this report is to increase awareness of this type of rickets (23).

## Data Availability Statement

The original contributions presented in the study are included in the article/supplementary material. Further inquiries can be directed to the corresponding authors.

## Ethics Statement

Ethics review and approval/written informed consent was not required as per local legislation and institutional requirements.

## Author Contributions

DA: first authorship. SH: last authorship. All authors contributed to the article and approved the submitted version.

## Conflict of Interest

The authors declare that the research was conducted in the absence of any commercial or financial relationships that could be construed as a potential conflict of interest.

## Publisher’s Note

All claims expressed in this article are solely those of the authors and do not necessarily represent those of their affiliated organizations, or those of the publisher, the editors and the reviewers. Any product that may be evaluated in this article, or claim that may be made by its manufacturer, is not guaranteed or endorsed by the publisher.
